# Signal-Based Self-Organization of a Chain of UAVs for Subterranean Exploration

**DOI:** 10.3389/frobt.2021.614206

**Published:** 2021-04-23

**Authors:** Pierre Laclau, Vladislav Tempez, Franck Ruffier, Enrico Natalizio, Jean-Baptiste Mouret

**Affiliations:** ^1^ Inria, CNRS, Université de Lorraine, LORIA, Nancy, France; ^2^ Université de Technologie de Compiègne (UTC), Compiègne, France; ^3^ Aix Marseille Université, CNRS, ISM, Marseille, France; ^4^ Université de Lorraine, CNRS, LORIA, Nancy, France; ^5^ Autonomous Robotics Research Centre, Technology Innovation Institute, Abu Dhabi, United Arab Emirates

**Keywords:** UAV (drone), RSSI (received signal strength indication), underground, subterranean, radio signal

## Abstract

Miniature multi-rotors are promising robots for navigating subterranean networks, but maintaining a radio connection underground is challenging. In this paper, we introduce a distributed algorithm, called U-Chain (for Underground-chain), that coordinates a chain of flying robots between an exploration drone and an operator. Our algorithm only uses the measurement of the signal quality between two successive robots and an estimate of the ground speed based on an optic flow sensor. It leverages a distributed policy for each UAV and a Kalman filter to get reliable estimates of the signal quality. We evaluate our approach formally and in simulation, and we describe experimental results with a chain of 3 real miniature quadrotors (12 by 12 cm) and a base station.

## 1 Introduction

Thousands of subterranean networks permeate the underground: caves, utility tunnels, abandoned mines, underground quarries, sewers, etc. These voids often need to be mapped and inspected, typically to ensure the safety of new buildings or tunnels, but also in case of obstruction or intrusions.

Robots would greatly help the inspection of these networks, which are often too confined for humans (e.g., sewer pipes) or too dangerous (caves, abandoned mines, collapsed buildings) (Morris et al., 2006). However, designing such robots is challenging. Firstly, they need very good off-road abilities, as the floor is typically uneven, with steep inclines, and sometimes flooded. Secondly, they have to be small enough to fit into tunnels that are often too narrow for humans. Overall, ground robots (wheels or tracks) are either too small to clear large obstacles or too big to enter narrow voids.

Flying robots (Unmanned Aerial Vehicles–UAVs) are a promising alternative to ground robots for subterranean operations (Freire and Cota, 2017; Preston and Roy, 2017; Rogers et al., 2017; Jones et al., 2019; Mansouri et al., 2019). As they fly, they are not impaired by obstacles or liquid on the floor. They can also easily fly over steep inclines, stairs, and even ladders. In addition, current quadrotors are cheap, well understood, and miniaturized. For instance, the Crazyflie is a research quadrotor that fits in a 12 × 12 cm square (rotors included) and weighs less than 40 g (Giernacki et al., 2017).

Unfortunately, small flying robots cannot carry a cable to stay connected with the operator, contrary to ground robots. Instead, they have to rely on a radio link, which works well in open air environments but is challenging in subterranean environments (Akyildiz et al., 2009; Khuwaja et al., 2018). In particular, a typical signal such as the one used by Crazyflie (2.4 GHz radio system) does not penetrate large amount of rocks and are heavily perturbed by metallic pipes. This means that the radio works underground only when there exists an unobstructed path between the emitter and the receiver: at each turn of a corridor, the signal is severely deteriorated or lost.

**FIGURE 1:**
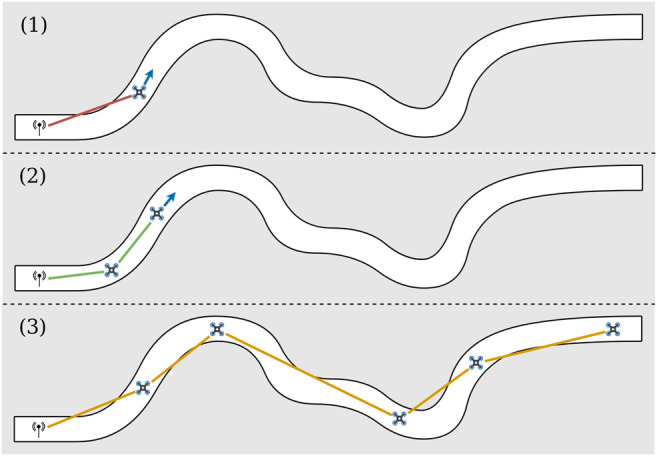
Illustration of the U-chain algorithm to maintain a communication chain in a tunnel. A human operator controls an explorer UAV in a tunnel. When the signal strength becomes too weak (1), a new autonomous UAV takes off and acts as a relay by finding the best relay position (2). The objective is the exploration of an environment at any distance, while maximizing the global signal quality (3).

In this paper, we introduce a distributed algorithm, called U-Chain (for Underground-chain), that coordinates a chain of UAVs, so that UAVs act as relays between an exploration drone and an operator (Figure 1). Our algorithm assumes that the UAVs are flying in a tunnel, but it only uses the measurement of the signal quality between each couple of UAVs and an estimate of the ground velocity based on an optic flow sensor. It does not need any localization system (e.g., a visual SLAM algorithm) and, as such, (1) it naturally adapts to the material and the topography of the tunnel and (2) it works on miniature platforms with limited computational power. For instance, our algorithm will place a relay at a corner (without knowing that it is a corner), but it will also increase the number of relays if the signal is highly perturbed in a specific zone for an unknown reason.

We show formally that maximizing the signal quality of a communication chain in a tunnel is equivalent to equalizing the signal quality between the UAVs, which leads to a simple positioning algorithm for the UAVs. Nonetheless, the signal quality depends on many parameters besides the distance between two UAVS, which results in a noisy value. To address this challenge, we introduce a Kalman filter that leverages optic flow-based measurements of the ground speed. We study the algorithm extensively in simulation and report experimental results on a chain of 3 miniature quadrotors and a base station.

## 2 Background

Most of the work about subterranean exploration has been focused on a single wheeled or tracked robot (Morris et al., 2006) that is coupled with a SLAM algorithm to build a 3-D map (Thrun et al., 2004). Single UAVs are, however, increasingly tested in mines and they show promising results (Freire and Cota, 2017; Preston and Roy, 2017; Jones et al., 2019; Mansouri et al., 2019). They have, for instance, been deployed by many teams during the DARPA Subterranean Challenge (https://www.subtchallenge.com/, 2020).

In parallel, many algorithms have been proposed for exploring unknown environments with a group of robots, with or without maintaining a permanent connection, and with or without a global coordination (Dorigo et al., 2013; Amigoni et al., 2017). To our knowledge, the vast majority of algorithms assume that the robots explicitly estimate their absolute or relative positions (Pei et al., 2010; Stump et al., 2011; Cesare et al., 2015; Amigoni et al., 2017; Nestmeyer et al., 2017), which they can share with the other robots, and sometimes know the environment beforehand (Hsieh et al., 2008; Stump et al., 2011). Using a positioning system makes sense in outdoor situations, in which the GPS signal is available, and indoor when each robot embeds a SLAM algorithm. Nevertheless, underground miniature UAVs cannot use GPS and they do not have the computational power and sensors needed for accurate SLAM. A notable exception for outdoor networks is the work of Hauert et al. (2009), which leverages an evolutionary algorithm to find a reactive strategy that maintains the network without positioning information.

Signal quality estimates are often considered too unreliable to solely drive the organization of the group of robots (Amigoni et al., 2017), in particular because it is influenced by many factors that are unknown from the robots and therefore not modeled (Amigoni et al., 2017; Khuwaja et al., 2018). Nonetheless, they were recently used in an exploration algorithm for the Crazyflie quadrotors (the same quadrotors as those used in the present work) (McGuire et al., 2019) for three purposes: (1) going back to the base station (by following the gradient of signal quality between the quadrotor and the base), (2) avoiding the other robots (by looking at the inter-quadrotor signal quality), and (3) adapting the exploration direction (by choosing directions that move away from the other quadrotors). This latter system shows that the signal quality can be useful, but it assumes that the base station can always communicate with the UAVs: the objective is not to maintain the communication, but to explore with a lightweight strategy based on reactive behaviors and a state machine.

In the present work, robots are assumed to be in a tunnel, which means they only need to decide if they go forward or backward to maintain the connectivity: this simplifies the coordination problem. In that particular situation, we hypothesize that the signal quality estimates can be sufficient to maintain a chain of communication. This kind of signal-based coordination was investigated in the particular case of subterranean tunnels by Rizzo *et al.* with wheeled robots (Rizzo et al., 2013). In their article, the authors analyze experimentally and theoretically the signal propagation in tunnels to obtain the general characteristic parameters; they describe a technique that uses these parameters to coordinate robots. While this approach is successful, it assumes a precise identification of the parameters of the radio signal before deployment, which we cannot obtain while exploring unknown environments. Moreover, the approach of Rizzo *et al.* slightly differs from ours in their objective: while they aim at keeping the quality greater than a minimum threshold, our algorithm maximizes the global quality of the connection.

## 3 Problem Formulation

We consider a chain of UAVs in a corridor without any positioning system. The general objective is to maintain a high-quality connection between each UAV and the next so that the first UAV of the chain can communicate with the last one. As the UAVs are in a corridor, they only need to decide whether to move forward or backward (the turning is managed by a separate algorithm); they base their decision on the estimates of the signal quality (RSSI—Received Signal Strength Indicator) with the previous UAV and with the next UAV in the chain.

Since the network is organized as a linear formation, a good way of estimating the global signal strength is by finding the relay link with the lowest chances of successfully transmitting the packet. We will call it the worst *bottleneck* in the communication chain. In this problem, this corresponds to maximizing the worst signal quality between two consecutive UAVs in the communication chain.

### 3.1 Notations

- A: 2D curve that describes the corridor geometry.
- $A=\left\{a_{0},\ldots ,a_{n}\right\}$: set of UAVs in the chain.
- $R\subset A,\;R=\left\{a_{1},\ldots ,a_{n-1}\right\}$: autonomous relay drones that physically position themselves between the head $H=a_{0}$ and the base $B=a_{n}$ to create a relay chain.
- $s_{\min}$: manually set threshold indicating whether the signal strength ensures a stable connection or not. After experiments with the Crazyflies, this value is set to 60.
- $P=\left\{x_{0},\ldots ,x_{n}\right\}$: set of curvilinear abscissa positions of the UAVs along the centerline of the tunnel starting at 0.
- $s\left(x_{1},x_{2}\right)$: function that returns the signal quality between the positions $x_{1}$ and $x_{2}$.

### 3.2 Assumptions

- All agents position themselves at the centerline of the tunnel, equidistant from each wall (this is achieved with the centering policy, Section 5).
- $\forall i,j\in {\left\{0,\ldots ,n\right\}}^{2},\;i>j,\;x_{i}\leq x_{j}$: all drones are positioned in the chain according to their index.
- The function $\left(x_{1},x_{2}\right)\mapsto s\left(x_{1},x_{2}\right)$ is defined only for $x_{1}\leq x_{2}$ and is continuous, that is, we expect by convention to have the position closer to the origin as first argument of *s*. A symmetric extension of *s* could be written as $\hat{s}:\left(x_{1},x_{2}\right)\mapsto s\left(\min\left(x_{1},x_{2}\right),\max\left(x_{1},x_{2}\right)\right)$.
- $\left(x_{1},x_{2}\right)\mapsto s\left(x_{1},x_{2}\right)$ is a decreasing function: if $\left[x_{1},x_{2}\right]\subset \left[{\tilde{x}}_{1},{\tilde{x}}_{2}\right]$ then $s\left(x_{1},x_{2}\right)>s\left({\tilde{x}}_{1},{\tilde{x}}_{2}\right)$ (that is, the signal quality decreases when UAVs move away from each other).
- The growth of *s* is bounded by *k* (i.e., it verifies k-Lipschitz continuity): ∃k>0 such that $\forall \left(x_{1},x_{2}\right)$ such that $x_{1}\leq x_{2}$, $\forall \varepsilon \in \left[0,x_{2}-x_{1}\right]$:
  •$s\left(x_{1}+\varepsilon ,x_{2}\right)\leq s\left(x_{1},x_{2}\right)+k\cdot \varepsilon$
  •$s\left(x_{1},x_{2}-\varepsilon \right)\leq s\left(x_{1},x_{2}\right)+k\cdot \varepsilon$
- We neglect the communication time (in our experience, our UAVs need less than 0.25 ms to send a packet, which is much faster than the overall dynamics of the chain of drones).

### 3.3 Objective Function

An optimal configuration is the solution of:

$$x^{*}=\underset{x_{i}}{\arg\max}\left(\underset{i}{\min}\left(s\left(x_{i+1},x_{i}\right)\right)\right)$$ (1)

that is, we search for the set of positions that maximizes the signal quality of the weakest link. This corresponds to a traditional “max-min” decision rule (Jaffe, 1981).

Claim: If the function *s* is continuous then the optimal configuration $x^{\text{*}}$ verifies:

$$\forall \;\left(i,j\right)\in \left\{0,\ldots ,n-1\right\}\;s\left(x_{i+1}^{*},x_{i}^{*}\right)=s\left(x_{j+1}^{*},x_{j}^{*}\right)$$ (2)

Proof:

The idea is that a configuration with unequal links can be improved by moving the UAV that is at the junction of the two unequal links by a step *ε* in the direction that improves the weakest of the two links. Thanks to the continuity of *s* there exists a step *ε* small enough so that the previously strongest link is deteriorated to a value that is still better than the previously weakest link. This new configuration is better than the previous one.

More formally, we proceed by contradiction and assume that there exists an optimal solution $x^{\text{*}}$ that does not verify (2) (i.e., $\exists i,s\left(x_{i+1}^{*},x_{i}^{*}\right)<s\left(x_{i}^{*},x_{i-1}^{*}\right)$, or $s\left(x_{i+1}^{*},x_{i}^{*}\right)<s\left(x_{i+2}^{*},x_{i+1}^{*}\right)$). Without loss of generality we can assume the former. We show, by descending recurrence on the number of links in $x^{\text{*}}$ that are of minimal quality, that there exists a better configuration.

If there is only one such link we denote the indices of the UAVs forming this link *j* and j+1. Thanks to the continuity of *s*, ∃ε>0,

$$s\left(x_{j+1}^{*},x_{j}^{*}\right)<s\left(x_{j+1}^{*},x_{j}^{*}-\varepsilon \right)<s\left(x_{j}^{*}-\varepsilon ,x_{j-1}^{*}\right)<s\left(x_{j}^{*},x_{j-1}^{*}\right)$$

If *j* was the head UAV (j=0) we can choose to move the UAV j+1 instead to get a similar result. As the link between *j* and j+1 was the only link with minimal quality, the new configuration given by $x_{k}=x_{k}^{*}$ if k≠j and $x_{j}=x_{j}^{*}-\varepsilon$ is a better configuration than $x^{\text{*}}$ because the worst link was improved without degrading too much the only other link that was changed: $s\left(x_{j},x_{j-1}\right)=s\left(x_{j}^{*}-\varepsilon ,x_{j-1}^{*}\right)$.

If there are more than one link, we can make a configuration change similar to the case described before to reduce the number of minimal quality links by one. We then start again with the new configuration until the configuration contains only one link with minimal quality and we can apply what is described above. Let *j* and j+1 be the indices of the UAVs forming one of those weakest links such that one its adjacent links (j−1,j) and (j+1,j+2) is not a link of minimal quality. Such a link exists because if all links with minimal quality were bordered by links whose quality is also minimal then all links would have the same quality. That would be in contradiction with our hypothesis about $x^{\text{*}}$. We assume that the link adjacent to (j,j+1) with a quality that is not minimal is (j−1,j). Thank to the continuity of *s*, ∃ε>0,

$$s\left(x_{j+1}^{*},x_{j}^{*}\right)<s\left(x_{j+1}^{*},x_{j}^{*}-\varepsilon \right)<s\left(x_{j}^{*}-\varepsilon ,x_{j-1}^{*}\right)<s\left(x_{j}^{*},x_{j-1}^{*}\right)$$

If the adjacent link whose quality was not minimal was (j+1,j+2) we can get a similar result by moving $x_{j}^{\text{*}}$ in the other direction, away from $x_{j+1}^{*}$. Thanks to this inequality that we get a new configuration *x* such that $x_{k}=x_{k}^{*}$ if k≠j and $x_{j}=x_{j}^{*}-\varepsilon$. This configuration possesses one less weakest link than $x^{\text{*}}$ because the two links that were changed now have a quality that is better than the worse quality of $x^{\text{*}}$ which was equal to $s\left(x^{\text{*}}j+1,x_{j}^{\text{*}}\right)$. Thus we have a new configuration *x* that is as good as $x^{\text{*}}$ but with one less weakest link.

By recurrence we can build a new configuration *x* that is better than $x^{\text{*}}$ which was supposed to be optimal. This contradiction proves that an optimal configuration cannot have links of unequal quality and has to verify Eq. 2 of the claim. □

These equality constraints (Eq. 2) define a necessary condition for an optimal configuration that we will later prove to also be sufficient. Please note that the fact that all the links’ qualities are equal does not imply that the distance between two successive UAVs is equal, because the link quality is affected by the environment (e.g., a turn or a different wall material).

We denote by $s_{eq}$ the link quality of configurations with equal links. We know that at least one exists because the algorithm introduced in Section 4 is proven to converge to equal links’ qualities. Moreover, since the function *s* is continuous, the function giving the minimal quality of a configuration is also continuous, the set of all configurations is compact (closed, bounded in finite dimension), thus there exists an optimal configuration.

Claim: Given the initial and end positions of the chain ($x_{0}=x_{t}$ and $x_{n}=0$) and the number of UAVs *n*, if the function *s* is a decreasing function (i.e. $\left[x_{1},x_{2}\right]\subset \left[{\tilde{x}}_{1},{\tilde{x}}_{2}\right]\;\Rightarrow \;s\left(x_{1},x_{2}\right)>s\left({\tilde{x}}_{1},{\tilde{x}}_{2}\right)$) then any configuration with equal links is a solution of the optimization problem stated in (1).

This result is not straightforward as we do not know much about the signal quality function *s*. In particular, it is straightforward to find examples of non optimal solutions with equal links’ qualities when *s* is *not* decreasing. For example, when the UAVs are in a u-shaped environment and the signal can traverse the walls, the optimal signal quality would be achieved by putting all the UAVs at the end of the U (closest to the starting point when we ignore the walls), but there are many sub-optimal solutions with equal signal qualities and a worst global quality.

To show that all solutions with equal links are optimal we show that there is only one solution with equal links.

Proof: (by contradiction). We assume that there exist at least two configurations *C* and $\tilde{C}$ with all equal links but a different signal quality for these links and same fixed position for first and last UAVs i.e.:

$$\{\begin{array}{c} \forall i<n\;s\left(x_{i+1},x_{i}\right)=s_{eq}\\ x_{0}=x_{t}\\ x_{n}=0 \end{array}$$ (3)

$$\{\begin{array}{c} \forall i<n\;s\left({\tilde{x}}_{i+1},\tilde{x_{i}}\right)=\tilde{s_{eq}}\\ {\tilde{x}}_{0}=x_{t}\\ {\tilde{x}}_{n}=0 \end{array}$$ (4)

$$and:\;\tilde{s_{eq}}<s_{eq}$$ (5)

We prove by descending recurrence on *i* that all $\tilde{C}$ UAVs positions ${\tilde{x}}_{i}$ are farther than those of *C*: ∀i<n, ${\tilde{x}}_{i}>x_{i}$.

We have $x_{n}={\tilde{x}}_{n}$ because the last UAV is fixed. As a consequence we have either

$$\left[x_{n},x_{n-1}\right]\subset \left[{\tilde{x}}_{n},{\tilde{x}}_{n-1}\right]or\;\left[{\tilde{x}}_{n},{\tilde{x}}_{n-1}\right]\subseteq \left[x_{n},x_{n-1}\right]$$ (6)

If the last option was true, because *s* is decreasing, we would have:

$${\tilde{s}}_{eq}=s\left(\tilde{x_{n}},{\tilde{x}}_{n-1}\right)\geq s\left(x_{n},x_{n-1}\right)=s_{eq}$$ (7)

that in contradiction with (Eq. 5), therefore $x_{n-1}<{\tilde{x}}_{n-1}$.

Now, if we assume that ${\tilde{x}}_{i+1}>x_{i+1}$ we cannot have ${\tilde{x}}_{i}\leq x_{i}$ as it would give that $\left[{\tilde{x}}_{i+1},{\tilde{x}}_{i}\right]\subset \left[x_{i+1},x_{i}\right]$ and thus ${\tilde{s}}_{eq}=s\left({\tilde{x}}_{i+1},{\tilde{x}}_{i}\right)>s\left(x_{i+1},x_{i}\right)=s_{eq}$, which is in contradiction with (Eq. 5), therefore:

$$\left({\tilde{x}}_{i+1}>x_{i+1}\right)\Rightarrow \left({\tilde{x}}_{i}>x_{i}\right)$$ (8)

This proves by recurrence that ${\tilde{x}}_{0}>x_{0}$ i.e. that *C* and $\tilde{C}$ have different position for the head UAV, which is in contradiction with the initial hypotheses. We can deduce from this proof that for a given initial and end positions, a given number of UAVs, there is only one configuration for which signal quality is equal on all links. As we proved before that the optimal solution as equal links, then any configuration with equal links is optimal because there is only one verifying that. Moreover, we showed in this proof that signal quality determines UAVs positions given number of UAVs and position of first and last UAVs. □

The optimization problem has been so far defined for a fixed number of UAVs. We also want to use the smallest possible number of UAVs to explore a given area. To do so, we modify the optimization problem to minimize the number of UAVs, keeping the signal quality equal between UAVs:

$$n^{*}=\arg\min n$$ (9)

subject to: (10)

$$\{\begin{array}{c} \forall i,j\in {\left\{0,\ldots ,n-1\right\}}^{2}\;s\left(x_{i+1},x_{i}\right)=s\left(x_{j+1},x_{j}\right)\\ \forall i\in \left\{0,\ldots ,n-1\right\}\;s\left(x_{i+1},x_{i}\right)\geq s_{\min}\\ x_{n}=0\\ x_{0}=x_{t} \end{array}$$ (11)

Enforcing the equality of all link qualities ensures that we still optimize the worst signal quality but here we also constrain to a minimal signal quality $s_{\min}$ to prevent signal loss along the chain: we proved previously (see Eq. 8) that if the number of UAVs, the start position, the end position and the function *s* are fixed, then $s_{eq}$ is fully determined; here, we fix the start and we authorize the addition of UAVs until $s_{eq}>s_{\min}$.

## 4 Positioning Algorithm

Our objective is to design a distributed algorithm that minimizes the number of UAV (Eq. 9) under the constraints defined by Eq. 11. We show that:

- Given a number of UAVs, an optimal distributed algorithm consists in equalizing the signal quality (Section 4.1) by moving each UAV forward or backward in the tunnel;
- An estimate of the signal quality can be obtained by filtering the RSSI with a 1-D Kalman filter that exploits an optic flow sensor (Section 4.2);
- New UAVs should take off when the link quality is below a given threshold (Section 4.3), so that only the minimum number of UAVs is flying.

In the following, we assume that:

- The head UAV can be moved forward or backward by the operator, but it can be forced to go backward or to stop (by overriding the command from the operator) to prevent a loss of connection;
- All the UAVs stay centered in the corridor thanks to the centering policy (Section 5).

### 4.1 Convergence to the Optimal Configuration

Since the UAVs move in a one-dimensional environment, the only decision to take at each step is either to go forward or backward along the only possible path. A reactive controller (Section 5) keeps each UAV at the centerline of the tunnel.

We assume that all the UAVs are in a sub-optimal configuration and that they need to converge to the optimal one. To equalize signal quality, each UAV compares the quality of its connection with the previous and with the next UAV in the chain, and moves toward the UAV with which it has the worst link, as described in algorithm (Eq. 12). By doing so, it improves the worst link quality and degrades the best one, moving them closer to equality. The speed at which the UAV moves is determined by the signal quality of its two neighboring links so that it does not move too fast (See Section 4.1), and, consequently avoid oscillations. In the following equation, *k* is the Lipschitz coefficient of *s* as detailed in Section 3.

$$\begin{array}{c} Let\;s_{d}=s\left(x_{i+1},x_{i}\right)-s\left(x_{i},x_{i-1}\right),\\ a_{i}\;moves\;by\;\varepsilon_{i}=\frac{s_{d}}{3k} \end{array}$$ (12)

Claim: The algorithm presented in Eq. 12 converges to a configuration where all links’ qualities are equal.

**FIGURE 2:**
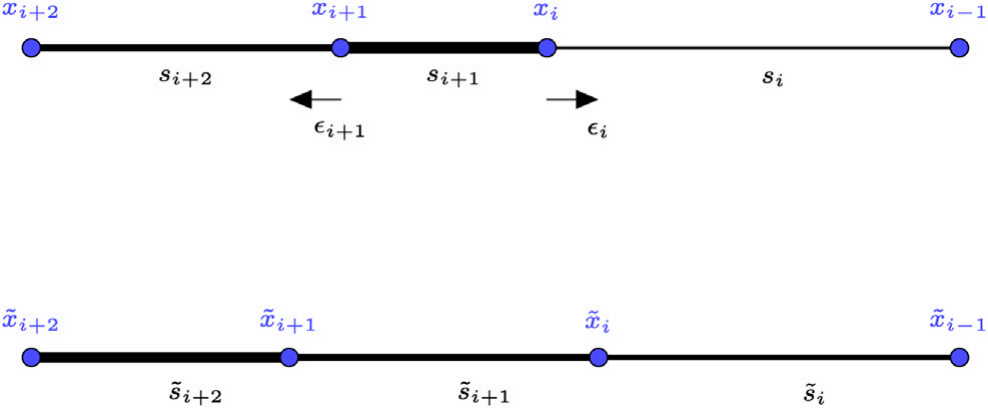
Illustration of one step of the positioning algorithm for the case of a strong link bordered by two weak links. The strength of a link is represented by its width.

Proof: In 3.3 we proved that unequal configurations were improved by moving by a small quantity *e* an agent at the junction of two unequal links. Here, the algorithm does that in a distributed fashion for each agent seeing unequal links. We want to ensure that the simultaneous contributions from all agents still work the same way. More precisely we want to see if the weakest of all links after each agent moved is stronger than the weakest of all links before the movement. To do that, we check that a strong link, that is weakened from both sides by the movement of agents, is still stronger than the previous weakest links, and that weakest links get stronger. We denote by $\varepsilon_{i}$ the movement of agent $a_{i}$, $s_{i}=s\left(x_{i+1},x_{i}\right)$ the link quality before the movement, $\tilde{x_{i}}=x_{i}+\varepsilon_{i}$ the position after the movement and ${\tilde{s}}_{i}=s\left(x_{i+1}+\varepsilon_{i+1},x_{i}+\varepsilon_{i}\right)$ the link quality after the movement (Figure 2).

First we prove that a strong link is not too weakened after one step. Let $s_{i-1},s_{i+1},s_{i}$ be 3 links in which $s_{i}$ is the strongest: $s_{i}>s_{i-1},s_{i+1}$. We have that:

$$\varepsilon_{i}=\frac{s_{i}-s_{i-1}}{3k},\varepsilon_{i+1}=\frac{s_{i+1}-s_{i}}{3k}$$ (13)

and

$$\begin{array}{c} s_{i}=s\left(x_{i+1},x_{i}\right)\\ =s\left(x_{i+1}+\varepsilon_{i+1}-\varepsilon_{i+1},x_{i}+\varepsilon_{i}-\varepsilon_{i}\right)\\ =s\left({\tilde{x}}_{i+1}+\left(-\varepsilon_{i+1}\right),{\tilde{x}}_{i}-\varepsilon_{i}\right)\\ \leq {\tilde{s}}_{i}+k\left(-\varepsilon_{i+1}+\varepsilon i\right)\\ \leq {\tilde{s}}_{i}+k\left(\frac{s_{i}-s_{i+1}+s_{i}-s_{i-1}}{3k}\right) \end{array}$$ (14)

because the assumption we made on *s* growth. As a consequence we have:

$$\begin{array}{l} {\tilde{s}}_{i}\geq s_{i}-k\left(\frac{s_{i}-s_{i+1}+s_{i}-s_{i-1}}{3k}\right)\\ \geq \frac{s_{i+1}+s_{i-1}+s_{i}}{3}\\ >\min\left(s_{i+1},s_{i-1}\right) \end{array}$$ (15)

This inequality confirms that the stronger link did not get weakened enough to have a quality lower that the quality of its neighbors before the move. It means in particular that it is still better than the worse link of the configuration.

For the second part, let $s_{i}$ be a link achieving minimum quality in the configuration. As $s_{i}$ is minimal, the two UAVs bordering it can only move to strengthen it. As a consequence, no weak link is made weaker. In addition, if $s_{i}$ is bordering a link that is not minimal, then it will be strengthened because the corresponding *ε* will not be 0. For weakest links that have also weakest links as neighbors, nothing changes at this step. In a non optimal configuration, at least one weakest link has a neighbor with a higher signal quality. As a consequence, the number of links with minimal quality decreases by at least one at each step or the last link of minimal quality improved. We have an increasing sequence of configurations bounded by the optimal configuration, therefore the algorithm converges. As the only configuration for which the algorithm is stable is the equal links configuration—that is also optimal—we can deduce that this algorithm converges to the optimal configuration. □

In order to ensure convergence, the algorithm needs to compute for each agent $a_{i}$ the distance to move $\varepsilon_{i}$. This value depends on the measure of signal quality and the Lipschitz coefficient *k* of *s*. However, the proof of convergence of our algorithm shows that if the agent $a_{i}$ moves by at most $\varepsilon_{i}$ in the correct direction then there is still convergence. Therefore, the measures of signal quality and the value of *k* need not to be exact and an upper bound can be sufficient.

Overall, the UAVs reach a consensus on link quality because the configuration that maximizes the weakest link is the configuration of equal links, which is unique. Proving the consensus using the dynamics of the system (Ren et al., 2007) would require to write an equation for the evolution of $s_{ii}$ at each step. To do that we would need the inverse of *s* (to express how $s_{i}$ changes when each agent $a_{i}$ moves by $\varepsilon_{i}$ as a function of previous $s_{i}$). However, if we assume that *s* is bilipschitz, we might be able to write two dynamic equations that describe the evolution of the $s_{i}$ at maximum and minimum speed. These two equation would bound the true evolution of the $s_{i}$ and it might be possible to write a second version of the convergence proof using tools from the consensus theory (Ren et al., 2007).

### 4.2 Received Signal Strength Indicator Filtering With an Optic Flow-Based Kalman Filter

The algorithm defined in Eq. 12 makes all its decisions on measurements of the link quality, which is measured using the RSSI. Unfortunately, these measurements are noisy and not as reliable as we could hope. In particular, the signal quality is affected by many factors besides the emitter-receiver distance, including the orientation of the UAV, the current motor speed, the radio activity of the other UAVs, beams on the ceiling, and so forth (Khuwaja et al., 2018; McGuire et al., 2019).

We therefore need to filter the RSSI to remove the high frequency noise. Using a simple moving average filter would not be sufficient as the estimation would have too much lag when the agents move towards or apart from each other.

Please note that we are trying here to minimize the time between the measurement (the RSSI changed) and the decision (how to move to improve the situation). We are here neglecting the time lags induced by radio transmission because (1) few messages are exchanged compared to the bandwidth (our Crazyflie UAVs can exchange data up to 1 Mbit), (2) less than 0.25 ms is required to send a packet and get a response, which is fast compared to the dynamics of the whole chain (in our experiments, the drones move at a maximum of 0.3 m/s). To get the best communication performance, we would need to take special care of optimizing the number of packets (in particular, the frequency of packet exchanges) and use a different radio channel for each communication (since each UAV needs to communicate with only 2 other UAVs at most, there is no need to occupy the radio channel).

Our main insights are that (1) we know that the signal quality is likely to decrease when two UAVs move away from each other, and (2) the relative speed of two UAVs can be measured at no computational cost with miniature optic flow sensors (Ruffier et al., 2003). In addition, the miniature Lidars onboard help the UAVs to fly at a nearly constant height, which means that measured optic flow is therefore proportional to the ground speed. Optic flow sensors can also work in low-light conditions (Mafrica et al., 2016). Recent commercial optic flow sensors are derived from optical mouse sensors (e.g., Pixart PMW3901MB) and weight only a few milligrams.

We incorporate this simple model in a 1-D Kalman filter to improve the signal quality estimation. To our knowledge, Kalman filters have never been used to improve the assessment of the signal quality in UAVs, in particular with optic flow sensors. Nevertheless, a few articles used RSSI and Kalman filters for localizing humans or phones by either assuming that the human does not move between two measurements (Bulten et al., 2016) or by combining RSSI and external sensors (Paul and Wan, 2009).

The filter considers that the signal quality decreases linearly with the relative velocity $u_{k}$:

$$r_{k}=r_{k-1}+Au_{k}+\varepsilon$$ (16)

$$z_{k}=r_{k}+\delta$$ (17)

where $r_{k}$ is the quantity to be filtered (the RSSI) and $u_{k}$ the system state (the relative speed between the agents) at iteration *k*. ε∼N(0,Q) is the intrinsic *signal noise* (which is assumed to be known) and *A* the impact of system state on the filtered quantity. $z_{k}$ is the observation of the estimated signal written as the true signal $r_{k}$ with a *measurement noise* δ∼N(0,R).

The update of the Kalman filter occurs in two steps. Firstly, the *prediction a priori* stage updates:

$$\begin{array}{l} {\hat{r}}_{k|k-1}={\hat{r}}_{k-1|k-1}+Au_{k}\\ P_{k|k-1}=P_{k-1|k-1}+Q \end{array}$$ (18)

${\hat{r}}_{k|k-1}$ is the prediction *a priori* of the value of $r_{k}$ based only on the previous measures of it up to time k−1 and the current relative speed $u_{k}$. It has yet to take the new measurement $z_{k}$ into account. ${\hat{r}}_{k-1|k-1}$ is the estimation *a posteriori* of the signal at time k−1. $P_{k|k-1}$ is variance *a priori* of this estimation.

We finally compute the *a posteriori* estimates with:

$$\begin{array}{l} K_{k}=P_{k|k-1}{\left(P_{k|k-1}+R\right)}^{-1}\\ {\hat{r}}_{k|k}={\hat{r}}_{k|k-1}+K_{k}\left(z_{k}-{\hat{r}}_{k|k-1}\right)\\ P_{k|k}=\left(I-K_{k}\right)P_{k|k-1} \end{array}$$ (19)

with $K_{k}$ being the Kalman gain, and ${\hat{r}}_{k|k}$ and $P_{k|k}$ the estimation of signal and its variance *a posteriori*.

In both simulated and real tests, we hand-tuned *A* to minimize estimation noise as well as the delay between the true RSSI and the estimate.

The pseudo-code for the U-chain algorithm, which combines the equalization of the links (Section 4.1) and the Kalman filtering (Section 4.2), is displayed on Algorithm 1. The algorithm essentially gets the RSSI measurements for the previous and next links, filters them, then ask the UAV to either go backward, forward, or stay at the same position, depending on the difference between the two filtered RSSI.

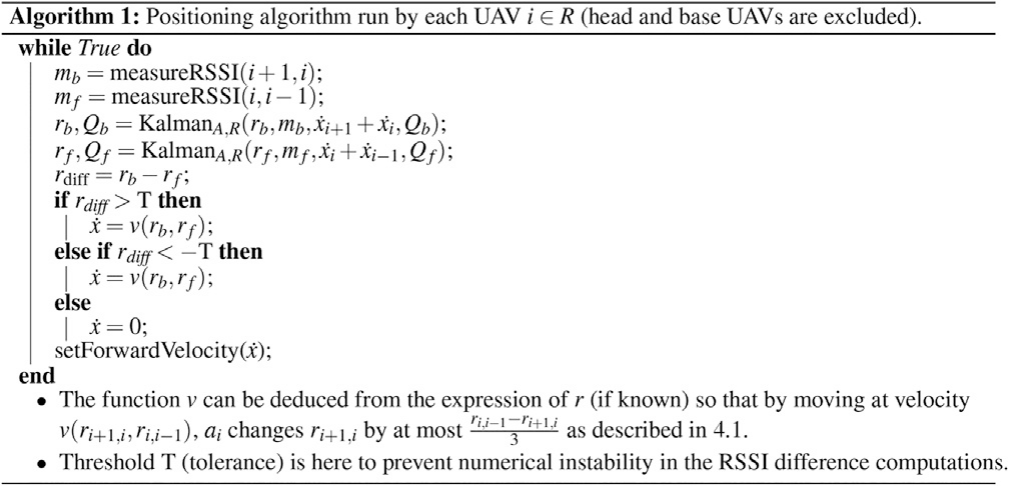

More precisely, the function $Kalman_{A,R}$ is the Kalman filter update described just before. $r_{b}$ and $r_{f}$ are the filtered value for RSSI for the UAV back and front in the chain; $Q_{b}$ and $Q_{f}$ are the uncertainty over these estimations. These values can be initialized with the first measurements $m_{b}$ and $m_{f}$ for $r_{b}$ and $r_{f}$, and with the value Q for $Q_{b}$ and $Q_{f}$.

### 4.3 Exploration With a Chain of UAVs

A straightforward use of Algorithm 1 is to move the first UAV (the head) forward in a tunnel and let the chain self-organize to maintain a stable connection. However, the link quality can drop below $s_{\text{min}}$ because the head is advancing, stretching the chain and decreasing the link quality of the links the chain $s_{eq}$. To ensure that communication is possible along the chain, if the base notices that the link quality with its neighbor is below the threshold $s_{\text{min}}$, a new UAV takes off and joins the chain.

We here assume that the algorithm that equalizes the links converges quickly enough so that links have converged when we make a new UAV take off. Thus, if the link quality between the base and its neighbor is below the threshold, it means that all the links’ qualities are below the threshold. If our assumption of fast convergence for the algorithm holds, the new UAV takes off only if the chain cannot be maintained with a link quality above $s_{\text{min}}$.

If no more relay UAVs are available to take off, the chain still equalizes the links’ qualities but may go under $s_{\text{min}}$. Additionally, the head UAV may move too fast and lose connection while exploring a new area (e.g., because of a sudden difficult propagation due to the local environment). A connection may still be possible but is not guaranteed. Therefore, all UAVs move automatically backwards when the connection to its predecessor is too weak or lost.

## 5 Centering Policy

**FIGURE 3:**
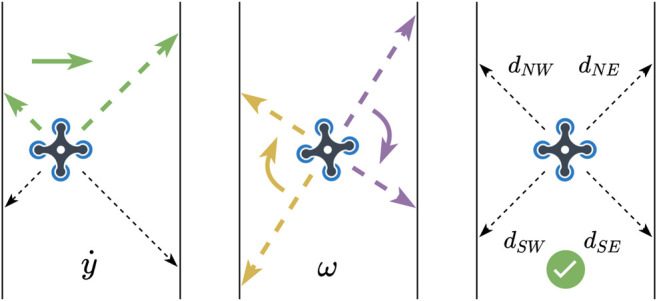
Main centering policy (Eq. 20). The quadrotors use the two front distance sensors ($d_{NW}$ and $d_{NE}$) to stay at the center of the corridor, and the difference between the sensors of the same side (e.g., $d_{NW}$ and $d_{SW}$) to control the yaw.

Our UAVs need to autonomously follow the explored tunnel, which can be achieved with a few miniature time-of-flight sensors (we use VL53L1x by ST Electronics). To do so, a reactive controller runs independently from the positioning algorithm to center each drone and follow the corridor turns. This policy uses four diagonal distance sensors to compute both the yaw and the lateral velocities (perpendicular to the wall). More precisely, at each iteration, in parallel with the calculation of $\dot{x}$ (Algorithm 1):

$$\{\begin{array}{c} \dot{y}=C_{t}\cdot \left(d_{NW}-d_{NE}\right)\\ \omega \;=C_{r}\cdot \left(d_{NW}-d_{SW}\right)+C_{r}\cdot \left(d_{SE}-d_{NE}\right) \end{array}$$ (20)

where $\dot{y}$ is the lateral velocity (the correction), $d_{NW},d_{NE},d_{NW},d_{SW}$ are the four distance sensors (see Figure 3), and both $C_{t}$ and $C_{r}$ are user-defined constants. Essentially, this policy uses the two front sensors to center in the corridor (the UAV is at the center of the corridor when both sensors have equal values), and the difference between the sensors from the same side for the yaw (the UAV is aligned with the walls when both sensors return the same value).

While surprisingly effective, this reactive policy is not always sufficient, in particular for 90 degrees turns. In this situation, the distance of one of the front sensors suddenly increases (e.g. $d_{NE}$ for a right turn), which creates a large distance difference between the two front sensors, and results in a dangerous *y* correction.

To mitigate this issue, we calculate the distances ratio of each pair of sensors that sense the left and right sides of the UAVs. If the difference percentage goes beyond a fixed value (40% after calibration tests), we consider that the wall is invalid and ignore it in the centering policy. When this happens, the drone automatically follows the remaining wall at a fixed distance by applying (for example, when the right wall is lost):

$$\{\begin{array}{c} \dot{y}=C_{t}\cdot \left(d_{NW}-D\right)\\ \omega \;=2\cdot C_{r}\cdot \left(d_{NW}-d_{SW}\right) \end{array}$$ (21)

with *D* being a preset distance. This additional policy makes it possible to explore wide rooms by following one side of the room, in addition to center in a tunnel.

## 6 Experimental Results

### 6.1 Simulation

#### 6.1.1 Main Assumptions

- The internal decision loop of each agent runs at 5 Hz to ensure that the algorithm will be easy to implement in the Crazyflies’ micro-controller.
- The UAVs communicate with simulated packet transmissions that are possible only if $s\left(x_{1},x_{2}\right)>s_{\min}$.
- The environment is 2-D.
- The distance sensors are simulated according to the environment, and the UAVs follow the navigation policy (Section 5).
- In these simulations, the communication is considered to be instantaneous.

#### 6.1.2 Signal Propagation Model

We model the RSSI according to models given by Vinogradov et al. (2018). The signal loss estimation is computed as:

$$RSSI\left(x_{1},x_{2}\right)=10\times \alpha \left(x_{1},x_{2}\right)\times \log\left(\left|x_{1}-x_{2}\right|\right)+\varepsilon$$ (22)

with α being the environment’s attenuation factor. When fixed, this expression corresponds to the *path loss* caused by air attenuation and is the main cause of signal degradation. Then, we include the *shadowing* phenomenon due to obstacles blocking the line of sight between the communicating agents by varying α, based on the environment. The value varies between 2 and 6 according to the amount of walls the segment between the agents passes through. ε∼N(0,B) is a Gaussian noise with a variance of 3.

Please note that RSSI $\left(x_{1},x_{2}\right)$ is defined as an increasing function, whereas we assumed so far that the signal quality decreases with the distance. As a consequence we choose $s\left(x_{1},x_{2}\right)=-RSSI\left(x_{1},x_{2}\right)$. As we use RSSI as a signal quality measure, and we observe that RSSI changes with time, we can wonder if it would be useful to introduce a time dependence in *s*. It is not necessary because the time contribution to RSSI comes from two sources, the first is some sensor noise, the second is indirect and comes from the position time dependence. That is why we decided to make *s* depend only on position and not time. Finally, we introduce a fixed 20% chance of losing a packet which is consistent with our experience with the Crazyflies.

In our experiments, we observed that the RSSI was degraded when the path between the two UAVs was passing through their motors (the motors are likely to interfere with the signal). As a consequence, the real RSSI depends on the relative yaw of the UAVs, in addition to the distance. We chose to neglect this effect in the simulation because it is not well documented and might be very specific to the type/position of the antenna of our UAVs (we preferred to use a well-validated model of signal propagation). In future work, this propagation model (Eq. 22) could be extended to take the yaw and the motor positions into account so that the RSSI increases when the direction of the target UAV is at (45,−45,225,−225) degrees from the source UAV, but this would require an extensive model identification to characterize the perturbation.

#### 6.1.3 Results

**FIGURE 4:**
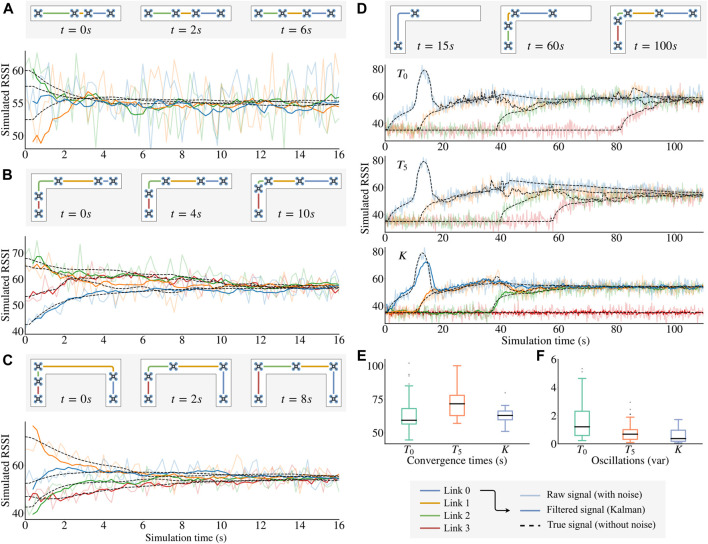
Simulation results. For each figure, the true signal (dashed line) is computed using Eq. 22 but without the Gaussian noise (this is unknown to our algorithm); the raw signal (light solid line) is the input of the U-Chain algorithm; and the filtered signal is the result of the Kalman filter used to take the movement decisions. **(A)–(C)** Convergence of Algorithm 1 in different environments (shown in the corresponding map thumbnails). *The UAVs place themselves at* “*strategic*” *positions*. **(D)** Comparison between three different signal processing methods: $T_{0}$ for no processing, $T_{5}$ for a tolerance of local signal difference of 5 and *K* for using a Kalman-filtered estimation of RSSI. **(E)** Comparison of the convergence times for 30 independent replicated runs on each of these three methods. **(F)** Variance of the UAV positions after convergence (30 replicates). *We observe that K gives a short convergence time (compared to* $T_{5}$ *) and less oscillation amplitude (compared to* $T_{0}$ *).*

We firstly checked that the chain successfully converges to stable positions and equalizes the signal quality when *n* UAVs are already flying in the air. To do so, we positioned the UAVs at random positions in the corridor for three different environments while fixing the position of the head. The results (Figures 4A–C show that the UAVs always converge to the desired solution, in spite of the noise on RSSI measurements. We confirmed this result by randomly generating 30 initial positions for 30 independent runs for each of the three environments: 100% of the runs converged to positions that equalize the RSSI.

We then evaluated the usefulness of the Kalman filter when the head drone is moving. We simulated a real exploration by placing all UAVs at the entrance of the corridor and launching the first UAV at the start of the simulation. The head then moves forward at a constant speed (0.2 m per second) before stopping after 50 s of exploration.

When using the raw signal as the entry for Algorithm 1 (Figure 4D, algorithm $T_{0}$), the system successfully converges as expected (Figure 4E). However, a single abnormally high value of RSSI caused by the noise can trigger the launch of an additional drone (see the red curve at 80 s) that is not needed to keep the true signal quality above $s_{\text{min}}$. Moreover, the variance of the UAV positions after reaching the final positions is particularly high (Figures 4F) which may cause stability problems when testing the system on real micro UAVs. Figures E and F only take the first three links’ qualities into account in order to ignore the non-repeatable launch of the forth UAV.

A simple way of trying to reduce these oscillations is to introduce a tolerance around the local quality equilibrium of each UAV before applying any movement (algorithm $T_{5}$). This indeed reduces the oscillations after convergence but unfortunately increases the convergence time, as small position corrections near convergence only happen when the noise makes the RSSI measurement exceed the tolerance value.

The Kalman filter improves the system performance on those two factors. The estimation greatly reduces the noise of the signal used in Algorithm 1 which leads to both a short convergence time and less oscillation.

### 6.2 Real-World Experiment

We designed a circuit that resembles a real corridor or pipe and respects the monotonic propagation constraint. The walls are made of thin paper boards for installation simplicity, and therefore do not block the signals in any way (to respect the monotonic propagation constraint, we therefore had to choose the shape of the tunnel carefully). However, these walls prevent the UAVs from taking the shortest path, which means that the signal quality follows a complex non-linear function along the tunnel. While this experiment does not fully capture an underground scenario, it makes it possible to validate the centering policy, the take-off decisions, and the overall maintenance of the chain.

**FIGURE 5:**
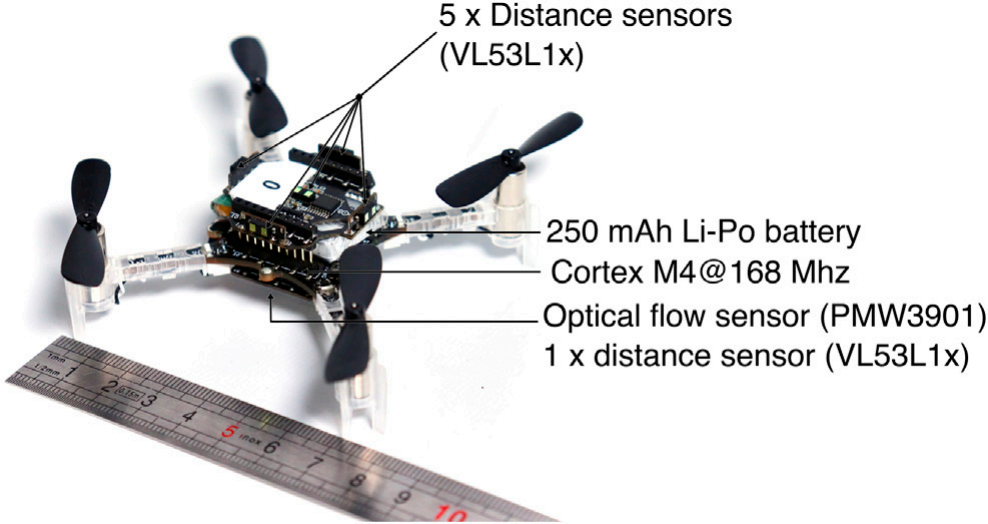
The Crazyflie 2.1 quadrotor (Giernacki et al., 2017). The quadrotor used in the real experiments (Figure 6) is about 12 cm by 12 cm (including the propellers). We use the “flow deck”, which adds an optical flow sensor and a distance sensor (pointing towards the ground) and the “Multi-ranger deck”, which provides 5 distance sensors (time of flight).

The micro UAVs used in our experiments are off-the-shelf commercial micro drones (Crazyflie 2.1 by Bitcraze, Figure 5) with two sensor add-ons (called “decks”). The first deck provides four time-of-flight distance sensors that detect obstacles up to 2 m (the detection angle is 27 degrees), which are used by the centering policy (Section 5). The second deck provides a fifth time-of-flight distance sensor that points downward, which is used to stabilize the UAV vertically. The same deck embeds an optical flow sensor, which helps stabilizing the UAV (by avoiding to drift) and provides the data for the ground speed estimate used in our algorithm (Section 4).

We added drone-to-drone (peer to peer) communication, as the original firmware only supported packet transmissions between the micro UAVs and a computer. The source code of the upgraded firmware code is available online (https://github.com/resibots/crazyflie-firmware/, branch “cavemod”).

**FIGURE 6:**
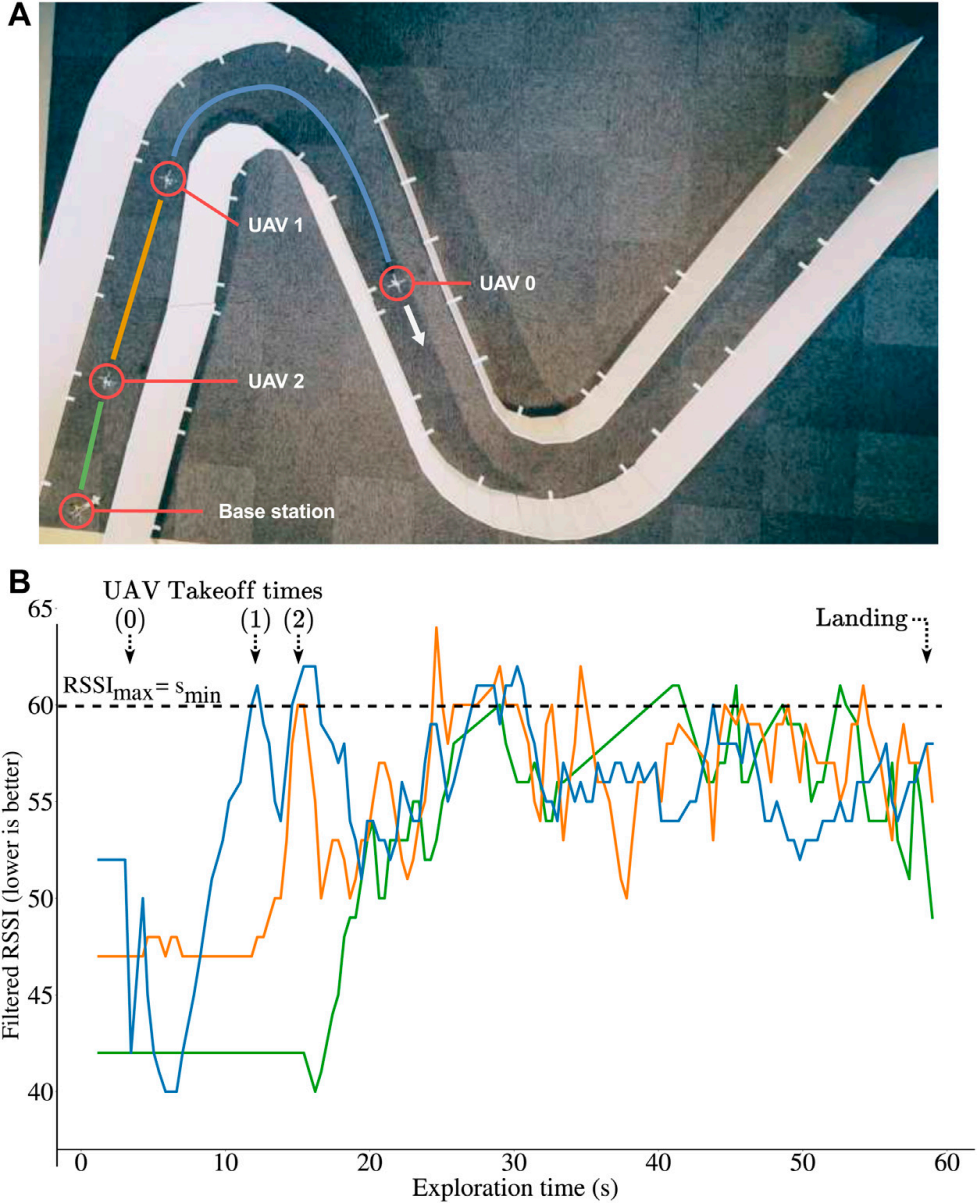
Experiments with 3 Crazyflie 2.1 quadrotors and a base station. The robots use an optic flow sensor and 5 time-of-flight sensors (altitude and centering). They communicate with a custom peer to peer protocol. **(A)** Environment used for the experiments. **(B)** Qualities of the radio links (RSSI) during exploration *in the real experiment* of panel A: after a few seconds, the quality of all the links are equal and below the threshold ($RSSI_{\max}=s_{\min}$). *A video is available with the submission.* Please note that the algorithm equalizes the signal qualities and not the distance, therefore it is not expected that the UAVs converge to being all equally distant.

In the experiments, all UAVs are initially positioned at the start of the corridor (Figures 6A) and are ready to take off when needed, except the last drone, which acts as a fixed base station. We launch the first UAV and manually control it with the keyboard keys (the pilot can only go forward or backward—the actual stabilization and centering is autonomous). The other UAVs then apply all presented algorithms in this paper to launch and relay the signal when needed.

Overall, the system performs similarly to the simulations (Figures 6B): all relays take off when the last active link reaches the fixed limit, and the UAVs successfully equalize the 3 links’ qualities until the end of the exploration. A video is available as Supplementary Material. At the end of the experiment, the robots are *not* equally spaced, which is expected because the algorithm equalizes the quality of the links and not the distances. This is an important feature because the link quality is what matters in this experiment, and they typically depend on many factors (including the materials and the presence of turns in the tunnel). In addition, the robots never fully stop (as seen on the video) because the signal quality, even filtered, is still imperfect.

## 7 Discussion

By combining a reactive positioning algorithm with a Kalman filter, the U-Chain algorithm can coordinate a chain of UAVs in a tunnel to maintain a high-quality connection while being light enough to be embedded on miniature UAVs. Since only the signal’s qualities are considered, the chain of UAVs adapts to any unexpected signal propagation, turns, radio perturbations, etc. While we did not consider intersections in this work, it is straightforward to store and transmit the direction choice made by the pilot who is controlling the head UAV, provided that a crude approximate of the position *x* in the tunnel is available (so that the following UAV can take the same decision).

The main hypothesis of the present work is that the quality of the signal decreases monotonically with the distance between an emitter and a receiver. This hypothesis is reasonable in an underground environment in which the signals never pass through walls: the signal is very unlikely to get better when the UAVs progress into a tunnel. However, this is often not the case in more general indoor environments, and in particular in modern buildings in which the radio signal can often pass through walls: in these cases, the signal can improve if there exists a path through the walls that is shorter than the physical path available (e.g., a door or a corridor). Future work will attempt to relax the monotony hypothesis while ensuring the convergence properties, in particular when the head UAV is moving.

The current algorithm assumes that the UAVs are moving in a single tunnel, without any branching. A natural extension is to modify it so that it can be used in more complex underground structures. In that situation, our vision is that the pilot of the head drone (or a exploration algorithm) has to decide which branch to follow, and the chain has to be maintained. If there are only two choices (left and right), then the centering policy needs to be slightly modified to make it possible to select which wall to follow (instead of centering between two walls or invalidating one of the walls in a big room); this modification is straightforward. The pilot then will be able to select which wall to follow, which will correspond to selecting the corridor. The positioning (forward or backward) algorithm should need no modification; however, the UAVs of the chain will also need to select which wall to follow when they reach an intersection. This would require them to know (1) the decision taken by the head UAV, which can be to transmitted by radio, and (2) their approximate position or a way to identify an intersection, which is challenging to obtain with a pure signal-based system. Solving this challenge might require the UAV to embed a place recognition system, a visual odometry system, or a SLAM system. If there are more than two branches from which to choose, then the pilot will need to drive the head drone to the branch he/she wants to explore and this decision needs to be replicated by the other drones of the chain, with an approach that needs to be determined.

The reported experiments, both in simulation and in reality, used “clean” (flat) walls for simplicity. However, our preliminary experiments with more rugged walls show that our centering policy is overall robust to deal with more complex environments. First, the centering policy is purely reactive, therefore ruggedness can only introduce small oscillations in yaw and translation. Nevertheless, the laser sensors that we use are designed to have a large field of view (27 degrees for the VL53L1X), which means that they tend to average the distances over a large surface (about 22 cm for a 50 cm distance). In other words, the surface is always smooth for the sensor. Second, the quality of the distance measurements is influenced by the light absorption of the material. However, if the material is the same on both walls (which is likely in a corridor), then the behavior will not change because the policy only computes relative differences (it equalizes the values); if they are different, then the UAV will not be centered anymore, but this will not change the overall behavior of the chain (what matters it that the UAV does not hit the wall). In our experience, the VL53L1X sensors are reliable for the vast majority of surfaces that we tested. In addition, complex, irregular radio absorption are invisible to the positioning algorithm, because it only looks at the empirical signal quality. As a consequence, we do not expect any issue with irregular absorption or diverse materials.

One the main limitations of tiny UAVs like the Crazyflie is their limited energetic autonomy (usually 5–15 min). An interesting research avenue is to extend our U-Chain algorithm to maintain the chain indefinitely by replacing UAVs with a low battery when needed; this could be useful to observe a place for a long time, or, for example, for keeping a radio link with a human (e.g., a stranded survivor of a mine collapse). A potential algorithm would be to continuously sort the UAVs so that the UAV with the lowest battery level would always be the closest to the base station and can be swapped with a fresh UAV when needed. This could be implemented in a distributed fashion by applying the “bubble sort” strategy: each UAV compares its battery level to the one of the previous one in the chain, and it swaps its position if its battery level is significantly lower. One of the main challenges of such an approach would be to implement the swap in a narrow tunnel without collision; another one is to maintain the connectivity during the swap, which will require substantial modifications of the positioning algorithm, as well as a temporary contractions of the chain and/or the addition of UAVs to compensate.

## Data Availability

The original contributions presented in the study are included in the article/Supplementary Material, further inquiries can be directed to the corresponding author.

## References

[B1] Akyildiz I. F. Sun Z. Vuran M. C. (2009). Signal propagation techniques for wireless underground communication networks. Phys. Commun. 2, 167–183. 10.1016/j.phycom.2009.03.004

[B2] Amigoni F. Banfi J. Basilico N. (2017). Multirobot exploration of communication-restricted environments: a survey. IEEE Intell. Syst. 32, 48–57. 10.1109/MIS.2017.4531226

[B3] Bulten W. Van Rossum A. C. Haselager W. F. G. (2016). “Human slam, indoor localisation of devices and users,” in First international conference on internet-of-things design and implementation (IoTDI), Berlin, Germany, April 6, 2016 (New York, United States: IEEE), 211–222. 10.1109/IoTDI.2015.19

[B4] Cesare K. Skeele R. Yoo S.-H. Zhang Y. Hollinger G. (2015). “Multi-UAV exploration with limited communication and battery,” in International Conference on Robotics and Automation (ICRA), Washington, United States, May 26–30, 2015 (New York, United States: IEEE), 2230–2235.

[B5] Dorigo M. Floreano D. Mondada F. Nolfi S. Baaboura T. Birattari M. (2013). Swarmanoid: a novel concept for the study of heterogeneous robotic swarms. IEEE Robot. Automat. Mag. 20, 60–71. 10.1109/mra.2013.2252996

[B6] Freire G. Cota R. (2017). “Capture of images in inaccessible areas in an underground mine using an unmanned aerial vehicle,” in Proceedings of the first international conference on underground mining technology, Sudbury, Canada, October 11–13, 2017. 10.36487/acg_rep/1710_54_freire

[B7] Giernacki W. Skwierczyński M. Witwicki W. Wroński P. Kozierski P. (2017). “Crazyflie 2.0 quadrotor as a platform for research and education in robotics and control engineering,” in 22nd international conference on methods and models in automation and robotics (MMAR), Międzyzdroje, Poland, Augest 28–31, 2017 (New York, United States: IEEE).

[B8] Hauert S. Zufferey J.-C. Floreano D. (2009). Evolved swarming without positioning information: an application in aerial communication relay. Auton. Robot 26, 21–32. 10.1007/s10514-008-9104-9

[B9] Hsieh M. A. Cowley A. Kumar V. Taylor C. J. (2008). Maintaining network connectivity and performance in robot teams. J. Field Robot. 25, 111–131. 10.1002/rob.20221

[B10] Jaffe J. (1981). Bottleneck flow control. IEEE Trans. Commun. 29, 954–962. 10.1109/tcom.1981.1095081

[B11] Jones E. Sofonia J. Canales C. Hrabar S. Kendoul F. (2019). “Advances and applications for automated drones in underground mining operations,” in Proceedings of the ninth international conference on deep and high stress mining, South Africa, June 29, 2019, 323–334.

[B12] Khuwaja A. A. Chen Y. Zhao N. Alouini M.-S. Dobbins P. (2018). A survey of channel modeling for UAV communications. IEEE Commun. Surv. Tutor. 20, 2804–2821. 10.1109/COMST.2018.2856587

[B13] Mafrica S. Servel A. Ruffier F. (2016). “Optic-flow based car-like robot operating in a 5-decade light level range,” in International conference on robotics and automation (ICRA), Stockholm, Sweden, May 16–21, 2016 (New York, United States: IEEE), 5568–5575.

[B14] Mansouri S. S. Castaño M. Kanellakis C. Nikolakopoulos G. (2019). “Autonomous MAV navigation in underground mines using darkness contours detection,” in International conference on computer vision systems, Thessaloniki, Greece, September 23–25, 2019, 164–174.

[B15] McGuire K. De Wagter C. Tuyls K. Kappen H. de Croon G. (2019). Minimal navigation solution for a swarm of tiny flying robots to explore an unknown environment. Sci. Robot. 4, eaaw9710. 10.1126/scirobotics.aaw9710 33137730

[B16] Morris A. Ferguson D. Omohundro Z. Bradley D. Silver D. Baker C. (2006). Recent developments in subterranean robotics. J. Field Robot. 23, 35–57. 10.1002/rob.20106

[B17] Nestmeyer T. Giordano P. R. Bülthoff H. H. Franchi A. (2017). Decentralized simultaneous multi-target exploration using a connected network of multiple robots. Autonom. Rob. 41 (4) , 989–1011. 10.1007/s10514-016-9578-9

[B18] Paul A. S. Wan E. A. (2009). RSSI-based indoor localization and tracking using sigma-point kalman smoothers. IEEE J. Sel. Top. Signal Process. 3, 860–873. 10.1109/jstsp.2009.2032309

[B19] Pei Y. Mutka M. W. Xi N. (2010). “Coordinated multi-robot real-time exploration with connectivity and bandwidth awareness,” in International conference on robotics and automation (ICRA), Anchorage, Alaska, May 8–10, 2010 (New York, United States: IEEE).

[B20] Preston R. P. Roy J. (2017). “Use of unmanned aerial vehicles to supplement conventional investigation methods for underground open void stability and mitigation,” in Proceedings of the first international conference on underground mining technology, Sudbury, Canada, October 11–13, 2017, 10.36487/acg_rep/1710_49_preston

[B21] Ren W. Beard R. W. Atkins E. M. (2007). Information consensus in multivehicle cooperative control. IEEE Control. Syst. Mag. 27, 71–82. 10.1109/MCS.2007.338264

[B22] Rizzo C. Tardioli D. Sicignano D. Riazuelo L. Villarroel J. L. Montano L. (2013). Signal-based deployment planning for robot teams in tunnel-like fading environments. Int. J. Robot. Res. 32, 1381–1397. 10.1177/0278364913501779

[B23] Rogers J. G. III Sherrill R. E. Schang A. Meadows S. L. Cox E. P. Byrne B. (2017). “Distributed subterranean exploration and mapping with teams of UAVs,” in Editors Pham T. Michael A. K. Ground/air multisensor interoperability, integration, and networking for persistent ISR VIII, California, United States, April 10–13, 2017 (Washington, United States: SPIE). 10.1117/12.2266316

[B24] Ruffier F. Viollet S. Amic S. Franceschini N. (2003). “Bio-inspired optical flow circuits for the visual guidance of micro air vehicles,” in Proceedings of the 2003 international symposium on circuits and systems (ISCAS’03), Bangkok, Thailand, May 25–28, 2003 (New York, United States: IEEE). 10.1117/12.498193

[B25] Stump E. Michael N. Kumar V. Isler V. (2011). “Visibility-based deployment of robot formations for communication maintenance,” in International conference on robotics and automation (ICRA), Shanghai, China, May 09–13, 2011 (Piscataway,NJ: IEEE), 4498–4505.

[B26] Thrun S. Thayer S. Whittaker W. Baker C. Burgard W. Ferguson D. (2004). Autonomous exploration and mapping of abandoned mines. IEEE Robot. Automat. Mag. 11, 79–91. 10.1109/mra.2004.1371614

[B27] Vinogradov E. Sallouha H. De Bast S. Azari M. M. Pollin S. (2018). Tutorial on UAVs: a blue sky view on wireless communication. J. Mob. Multimed. 14, 395. 10.13052/jmm1550-4646.1443

